# Constraint-based stoichiometric modelling from single organisms to microbial communities

**DOI:** 10.1098/rsif.2016.0627

**Published:** 2016-11

**Authors:** Willi Gottstein, Brett G. Olivier, Frank J. Bruggeman, Bas Teusink

**Affiliations:** Systems Bioinformatics, Amsterdam Institute for Molecules, Medicines and Systems, VU University Amsterdam, De Boelelaan 1087, 1081 HV Amsterdam, The Netherlands

**Keywords:** microbial communities, community flux balance analysis, genome-scale stoichiometric models, metabolic interactions, growth strategies, derivation of flux balance analysis

## Abstract

Microbial communities are ubiquitously found in Nature and have direct implications for the environment, human health and biotechnology. The species composition and overall function of microbial communities are largely shaped by metabolic interactions such as competition for resources and cross-feeding. Although considerable scientific progress has been made towards mapping and modelling species-level metabolism, elucidating the metabolic exchanges between microorganisms and steering the community dynamics remain an enormous scientific challenge. In view of the complexity, computational models of microbial communities are essential to obtain systems-level understanding of ecosystem functioning. This review discusses the applications and limitations of constraint-based stoichiometric modelling tools, and in particular flux balance analysis (FBA). We explain this approach from first principles and identify the challenges one faces when extending it to communities, and discuss the approaches used in the field in view of these challenges. We distinguish between steady-state and dynamic FBA approaches extended to communities. We conclude that much progress has been made, but many of the challenges are still open.

## 1. Introduction

Microbial communities are ubiquitous and of great interest for biotechnological applications [1,2], health [3–6], food production [7] and environmental studies [8]. Key questions in the field of microbial ecology are: ‘Who are the community members?’, ‘What are they doing?’, ‘How do they interact?’ and ‘What functionality arises from these interactions?’. Metagenome sequencing is rapidly answering the first question [9–12], and this makes the other questions all the more pressing. Great strides have been made in analysing metabolic interactions between the members of microbial communities [13–17]. Despite this progress, the principles that shape the structure of microbial communities remain largely elusive, making it challenging to understand, describe, predict and (ultimately) design communities with particular behaviour.

Here, we review modelling approaches that take genome information as the primary input and aim to predict community behaviour from a metabolic perspective. Thus, we do not discuss interactions between microorganisms that have no direct metabolic component, such as physical interactions or signalling processes (quorum sensing, for example). The approaches we describe are all constraint-based, stoichiometric, modelling methods for metabolic networks. The associated models are essentially mappings of genes to metabolic enzymes, the reactions of which form a (genome-scale) metabolic network where the edges and nodes represent (enzyme-catalysed) reactions and metabolites, respectively.

Constraint-based modelling has been developed for sequenced microbes in monoculture, and many methods have been developed to reconstruct, query and predict the metabolic capacities of a species based on its genome sequence [18–24]. The most widely used method is flux balance analysis (FBA) [25,26]. Given a biologically relevant objective function and constraints on input/output rates (e.g. glucose consumption, carbon dioxide excretion), FBA predicts steady-state flux distributions that optimize the chosen objective—typically biomass formation, but also multi-dimensional objectives exist [27–29]. Under the right premises, to be discussed later, these predictions are often surprisingly accurate—which is remarkable considering that these models do not take kinetic information into account [24]. When metagenome sequencing became feasible for entire communities [9–12], the development of constraint-based stoichiometric modelling approaches for microbial communities has become attractive.

As we explain later in more detail, the results of FBA-type approaches mainly depend on (i) the quality of the reconstructed metabolic model, including the biomass composition of the associated microorganism and the assumed gene–protein reaction rules, (ii) the chosen objective function, (iii) the considered constraints for exchange and intracellular rates, and (iv) the considered nutrients that can be used for biomass and product formation. If FBA is extended to analyse metabolism on a community level, then additional layers of complexity are added to all four of these points.

With respect to reconstructions, despite the progress that has been made in automating the reconstruction of metabolic models on the genome scale [30–34], model reconstruction and manual curation is still a very time-consuming process. In particular, detailed biomass compositions are available only for a few microbial species, and their experimental determination requires the isolation and culturing of the species. If one misses physiological characteristics, e.g. certain auxotrophies or transport reactions in the reconstruction process, then it will have a considerable impact on the predicted interactions between organisms.

Once a genome-scale model has been reconstructed, it is essential for community modelling that it can be combined with other models in a meaningful way. The construction of community models is complicated by the fact that, in most cases, such models will have been created by different authors and research groups. Consider, for example, that all models should be encoded to be readable by the same simulation software; model components (reactions, metabolites, flux capacity constraints) must be unambiguously annotated such that they can be used to link models together and community models themselves should be reproducible and exchangeable. Fortunately, with regards to model interoperability, the constraint-based modelling community has recently adopted a standard model encoding format that allows for efficient model exchange [35]. However, the problem of seamless integration, when using models collected from different sources, currently remains open.

Another difficulty is to define a community objective function in both biological and mathematical terms; typically, a combination of the objective functions of individual species is optimized but the exact formulation can differ [15,17,36–39]. This question touches upon fundamental questions in evolutionary biology about group selection versus selection at the level of individuals.

Regarding exchange rates, at a monoculture level, exchange rates can be calculated from extracellular concentration data, and intracellular fluxes can be estimated using, for example, ^13^C-labelling experiments [40,41]. Such data can then be used as constraints for FBA. Both become much more complicated on a community level; it is not trivial to determine individual contributions of microbial species to measured net fluxes of extracellular compounds, making it difficult to define appropriate constraints on the species level. There are isotopic labelling methods for community-level quantification of fluxes, and metatranscriptomics and metaproteomics data may be translated to flux constraints under simplifying assumptions [42–48], but these have not reached a sufficiently quantitative level yet.

**Figure 1.**
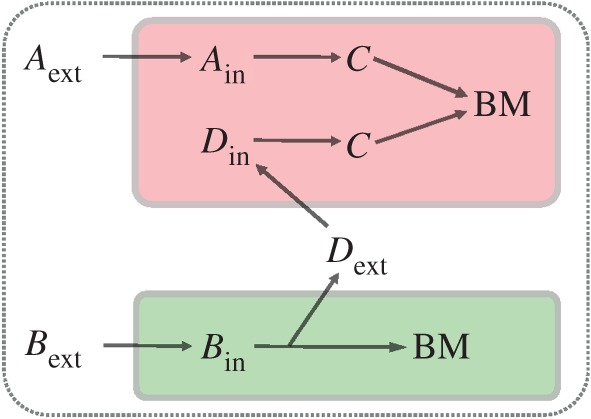
Growth potential depends on the composition of the medium and the metabolic capabilities of members in the community. Organism 1 (red) can only use *A*_ext_ that can be converted into a precursor of biomass, *C*. If it grew alone, then it could not make use of *B*_ext_. When organism 2 (green) is present, which is capable of using *B*_ext_ and thereby excreting a growth coupled compound *D*, organism 1 can increase its biomass production.

On a single-species level, the growth potential of organisms is directly determined by the composition of the medium. This is not so on a community level where the available resources and the metabolic capabilities of other community members determine the metabolism and growth behaviour of particular species. Even if species cannot use certain compounds available in their environment directly, they might be able to use those indirectly through the metabolic activity of other members in the community, as illustrated for a minimal example in figure 1. Interactions between species are therefore context and medium dependent [49–54]. Thus, at the moment, models are required to predict and quantify these (hidden) metabolic interactions between microbial species, given different environmental conditions and objectives, as we can presently not measure them.

In this review, we discuss the several methods that have been developed [14,15,17] to use FBA and variants thereof on a community level. The first work using genome-scale models for a microbial community was published in 2007 [36]. Since then, it has been used to estimate interspecies fluxes as well as intracellular flux distributions [36], to classify metabolic interactions [13], to predict the compositions of media that induce interactions between members of a community [55], to predict optimal relative biomass abundances [39] and many more, most of which are summarized in [17]. We view these studies in the light of the complications that we have identified. For a thorough understanding of these issues, however, we first need to derive the classical FBA approach from its basic principles before we move on to the specifics of community FBA (cFBA).

## 2. Basic principles and assumption of single-organism flux balance analysis: the foundation of microbial-community flux balance analysis

The foundations of classical FBA were developed in the 1980s [56–58] and today it is one of the most used methods to study metabolism [24–26]. By making assumptions about an objective function (typically biomass formation) and using information about transport fluxes (e.g. sugar uptake, product formation) and intracellular rates, FBA can be used to determine flux distributions that optimize the respective objective; kinetic parameters are not required. FBA and all of its variants rely on a metabolic steady state. Hence, it strictly applies only to cell populations displaying balanced growth, i.e. cells in batch culture growing in the exponential phase, or cells cultured in a chemostat, where the concentration of each metabolic intermediate and the growth rate are constant.

The rationale of FBA is as follows: one considers all metabolic reactions in the metabolic network, including those reactions leading to the macromolecular components of cells such as proteins, RNA, lipids and DNA (or their respective building blocks) and one that specifies the macromolecular component composition of cells, using an artificial biomass-forming reaction. Next, one demands a metabolic steady state, which then directly leads to a constant rate at which new biomass is synthesized. Hence, the balanced growth condition is modelled. To make this explicit and facilitate the discussion of modelling microbial communities, in the following classical FBA is derived from basic principles.

The starting point of this derivation is the definition of the concentration *c_i_* of a metabolic intermediate *i* in the metabolic network with *c_i_* = *n_i_*/*V*, where *n_i_* is the number of molecules *i* and *V* is the cell volume. *V* and *n_i_* are defined for the entire population of cells; *V* is the total cell volume and *n_i_* is the total amount of molecules *i*. Owing to growth, both variables are time dependent, and the temporal change of *c_i_* is given by applying the chain rule for differentiation2.1
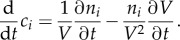
When the volume is constant, i.e. *∂V*/*∂t* = 0, the concentration change is proportional to the change in the number of molecules. When the number of molecules is fixed, ∂*n_i_*/*∂t* evaluates to 0 and *c_i_* can be increased when the volume is decreased and vice versa. At steady state, the temporal change of *c_i_* is 0, i.e. (d/d*t*)*c_i_* = 0. This yields for equation (2.1)2.2
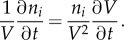
Multiplying equation (2.2) by *V*/*n_i_* allows us to define the specific growth rate *μ* at steady state as2.3
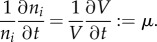


Rewriting equation (2.2) by using equation (2.3) illustrates that the synthesis of new molecules equals their dilution by (volume) growth at steady state2.4
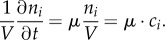


At a constant volume (so in the absence of volume growth), the rates of the reactions *v_j_* occurring in metabolism relate to equation (2.4) in the following way:2.5
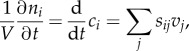
whereby *s_ij_* are stoichiometric coefficients which are positive/negative if metabolite *i* is produced/consumed in reaction *j*. Equation (2.5) means that the temporal change of *c_i_* is determined by fluxes *v_j_* that can either produce or degrade metabolite *i*. The vectorized version of equation (2.5) then reads2.6
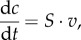
where *c* is the vector of concentrations, *v* is a vector of rates and *S* is the stoichiometric matrix with *m* rows and *r* columns representing metabolites and reaction rates, respectively. It contains the stoichiometric coefficients *s_ij_*.

### 2.1. Biomass and growth dilution

In FBA, one now defines two classes of metabolites that appear in the stoichiometric matrix *S*: molecules that are required for biomass formation (metabolic end-products such as DNA, RNA, protein, membranes or their respective building blocks) and intermediates which are not. These two classes are treated differently regarding the dilution by growth (equation (2.4)). It is neglected for intermediates as the metabolic rates of intermediate conversions are assumed to be much higher in value than *μ* · *c_i_*. For biomass components, however, dilution by growth is taken as their main sink, and, therefore, equations (2.4) and (2.5) become2.7
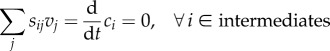
and2.8
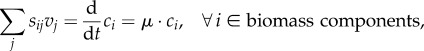
which means that intermediate concentrations do not change over time, i.e. their production rates equal their consumption rates, whereas biomass components accumulate exponentially with rate *μ*, reflecting the exponential increase of biomass. In stoichiometric models, the fluxes *v_j_* are usually expressed in mmol (h · g)^−1^ and growth rate *μ* has the unit g (g · h)^−1^ reflecting the increase of biomass per biomass per hour. Considering this, one sees that *c_i_* has the unit mmol g^−1^ (equation (2.8)). Hence, *c_i_* is the amount of biomass component *i* per gram of biomass. A biomass-forming reaction *v*_biomass_ can be defined as2.9
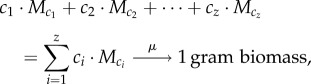
where 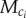 represents one of the *z* biomass components (dimensionless) and the coefficients *c_i_* the contribution of biomass component *i* to 1 g of biomass with unit mmol g^−1^.

**Figure 2.**
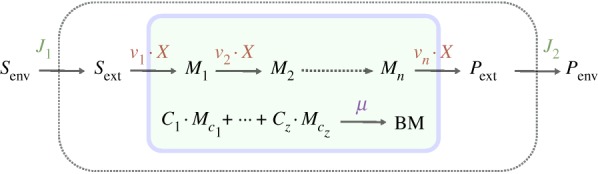
Illustration of the different rates in FBA. One distinguishes environment fluxes *J_i_* with unit mmol h^−1^, specific rates *v_i_* with unit mmol (h · g)^−1^ and the organism's specific growth rate in h^−1^. *X* denotes the organism's biomass in unit *g*, 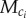 are components required for biomass formation and their respective contribution to 1 g of biomass is denoted by *c_i_* with unit mmol g^−1^.

Summarizing, FBA involves the following mass-balance constraints, with the rates *v_j_* as unknowns:2.10
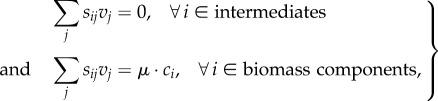
or in a more compact form2.11
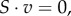
whereby the flux vector *v* contains *v*_biomass_ and the stoichiometric matrix *S* a corresponding column that contains 0s in rows that correspond to intermediates and the factors *c_i_* in rows that correspond to biomass components. So-called boundary species (*S*_env_ and *P*_env_ in figure 2) do not appear in the stoichiometric matrix; they just serve as parameters and do not have any influence on the actual simulations. In some model formulations, they are omitted, and the environmental fluxes *J* are expressed as ‘hyperspace’ reactions. The net units on the left- and right-hand sides of the relations in equation system (2.10) are per unit ‘gram cells’. So they remain valid when the biomass increases owing to growth. These relations are therefore the balanced growth condition that was mentioned earlier. The rate of biomass increase equals d*X*/d*t* = *μ* · *X* with *X* as biomass in gram cells. Biomass therefore does not need to be considered explicitly in FBA on monocultures. This is different for communities, as we see!

### 2.2. Flux distributions have variability

As there are typically more unknown fluxes than metabolites, the linear system defined in equation (2.11) is usually underdetermined. The solution space can be reduced (i) by constraining individual rates with so-called capacity constraints, using information about reversibility of reactions and constraints based on experimental data, such as uptake rates, and (ii) by optimizing a particular objective as, for example, *v*_biomass_, using linear programming. The optimization problem in FBA can be expressed in a compact form as follows:2.12
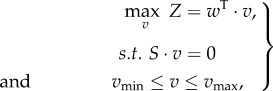
where *Z* represents the objective function and is expressed as a linear combination of fluxes *v* with weights contained in vector *w* and has been discussed extensively in [27,28]; typically, growth rate is maximized, thus *Z* = *v*_biomass_. The *v*_min_ and *v*_max_ contain the lower and upper bounds of the rates; if a reaction is irreversible, then the corresponding lower bound is set to 0.

Usually, multiple flux distributions exist that optimize the respective objective function. The flexibility of individual fluxes can be examined by using flux variability analysis (FVA), where each flux is minimized and maximized, using the optimal value of the objective function as an additional constraint [59–62]. FVA can be formulated as follows:2.13
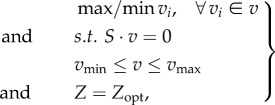
whereby *Z*_opt_ is determined, using FBA. More advanced methods exist to study all the alternative optimal flux distributions [63,64].

#### 2.2.1. A closer look at the units in classical flux balance analysis

In FBA, one can distinguish three different rates, which are depicted in figure 2: environmental (exchange) fluxes *J* with unit mmol h^−1^, specific rates *v* with unit mmol (h · g)^−1^ and a biomass-forming reaction *v*_biomass_ occurring at the specific growth rate *μ* which has the unit h^−1^. In the following, we explain how these different rates are connected, using the small example system from figure 2.

The external metabolite, *S*_ext_, is made available at a rate of *J*_1_(*t*) and is consumed by the organism at a rate of *v*_1_ · *X*(*t*). The temporal change of *S*_ext_ is therefore given by2.14
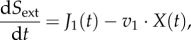
where *X*(*t*) denotes the biomass of the organism. In most FBA formulations, *S*_ext_ is also balanced by uptake and exchange reactions, and because the biomass increases exponentially the steady-state solution to equation (2.14) must read
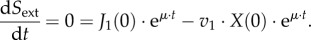
Normalizing this expression with respect to the total biomass given by
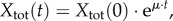
one obtains for the specific rate *v*_1_
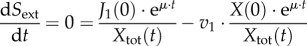

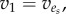
where2.15
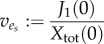
is the *specific* exchange flux with the environment. 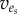 has the unit mmol (h · g)^−1^ consistent with the units of the remaining rates.

For all internal metabolites, the biomass cancels out at steady state, as shown here for metabolite *M*_1_2.16
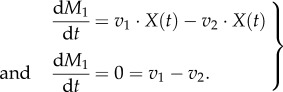
Note that this is not the usual way that FBA and the role of exchange fluxes are presented: rather, exchange fluxes *v*_e_ are often ‘just’ assigned for every external metabolite under the premise of fixed external metabolite concentrations in steady state. Yet, strictly speaking, *S*_ext_ can only remain constant under exponential growth if the feeding rate increases exponentially with biomass. It illustrates an important point often taken for granted: in FBA, we fix some input flux(es) through capacity constraints. In real systems, this can be achieved in different ways. In fed-batch growth, the feeding rate of *S*_ext_ is indeed increased exponentially to keep its concentration constant during exponential growth. In the chemostat, we use dilution to achieve a steady state of biomass and nutrient levels; in normal batch growth, we define a region where the inevitable changes in external metabolites do not affect the uptake (and production) rates. For substrates taken up, this implies saturation—or nutrient excess. As we see, a clear distinction of the units of the different fluxes will be essential to understand the FBA approaches applied to communities.

#### 2.2.2. Limitations of single-organism flux balance analysis

FBA has been proven to be a useful tool in several contexts, such as strain design [65–67], the outcome prediction of evolution studies [68,69] and gene deletion studies [70–72] (many more can be found in various reviews and references therein [24–26]). However, because of its stoichiometric nature, and because it uses optimization, FBA comes with inherent limitations. Most prominently, one should be aware of the fact that FBA predicts flux distributions that provide the maximal yield on the limiting nutrient, and does not actually predict kinetic rates. If one sets the uptake rate of a nutrient, e.g. *v*_glucose_, to a certain value and *μ* is maximized by finding optimal values of the remaining *v_j_*, then the fluxes that maximize the ratio *μ*/*v*_glucose_ are found. This expression is defined as a yield, *Y*_biomass/glucose_, with unit grams of biomass per mol glucose. While there are examples where the predicted flux distributions are in good agreement with experimental data [68,69,73], there is poor agreement under conditions of nutrient excess that favour low-yield strategies such as fermentation [74–76].

As mentioned earlier, owing to the assumption of time-invariant extracellular conditions, classical FBA can only be applied to chemostat cultures and cells in batches that grow exponentially, i.e. populations that display balanced growth. External fluctuations of metabolite concentrations cannot be incorporated. Furthermore, owing to the linear nature of the system, FBA neither allows absolute concentrations to be predicted nor incorporates saturation effects. In addition, regulatory effects cannot be examined. To overcome some of the limitations, classical FBA has been extended in recent years [77–84]. The most exciting extensions from a community point of view are dynamic FBA (dFBA) [85]—discussed in more detail later—and very recent methods that couple metabolic fluxes to enzyme synthesis costs, thus allowing growth-rate-dependent switches in metabolic fluxes according to the principle of limited resource allocation [82–84,86,87]. The latter approaches have not been used in community modelling as far as we are aware, but show much more realistic behaviour than classical FBA.

#### 2.2.3. Summary of the main assumptions for classical flux balance analysis

FBA allows steady-state flux distributions to be determined given a genome-scale stoichiometric model, flux capacity constraints (e.g. sugar uptake) and an objective function (e.g. growth rate). One distinguishes two classes of relevant metabolites for which different mass-balance constraints apply: intermediates for which dilution by growth is not considered and biomass components for which growth dilution is the main sink (equations (2.7), (2.8), (2.10)). There are three different kinds of rates: environmental exchange fluxes with unit mmol h^−1^, specific rates with unit mmol (h · g)^−1^ and a biomass-forming reaction occurring at the specific growth rate *μ* which has the unit h^−1^ (figure 2). In classical FBA, biomass does not have to be taken into account explicitly when uptake rates are calculated (§2.1) which is a major difference from FBA on the community level as discussed in the following sections.

## 3. Steady-state community flux balance analysis

In Nature, microorganisms usually do not occur in monocultures but are rather organized in communities where they interact in various ways [88]. Until now, it has remained a great challenge to qualify and quantify metabolic interactions between community members and to understand how these influence the community structure and its dynamics. For this purpose, several approaches have been developed [14,15,17], one of which is FBA on a community level where stoichiometric models of different species are connected [14,17,36,89]. Such a meta-species network allows for an analogue mathematical representation as described for classical FBA. The choice of an appropriate objective function, however, is even less obvious on the community level than it is for monocultures as there has been a long-standing debate on which level natural selection actually occurs [90–97].

Nevertheless, in brave attempts to use the time-invariant constraint-based formalism for communities, several expressions for an objective function have been proposed, such as linear combinations of individual species objectives [36], a community growth rate meaning that all species grow at the same rate [39] as well as a bilevel objective function where individual as well as community objectives are taken into account [37]. We now extend the mathematical formulation of classical FBA to describe the steady-state growth of communities.

**Figure 3.**
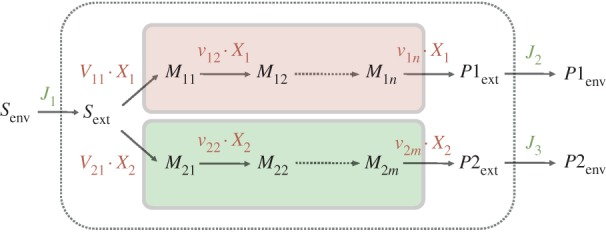
Two organisms live in the same environment competing for a substrate *S*_ext_. *X_i_* represent their respective biomass, *J_i_* are environment fluxes with unit mmol h^−1^ and *v_i_* are specific rates with unit mmol (h · g)^−1^. Internal metabolites are denoted by *M_ij_*.

For this, a community is considered that consists of only two members, as depicted in figure 3; a more generalized derivation can be found in [39].

First, the steady-state concentration of the external metabolite *S*_ext_ is considered (the same formalism applies to *P*1_ext_ and *P*2_ext_). *S*_ext_ is made available by an environmental flux *J*_1_ and taken up by both organisms. These uptake rates are proportional to their biomass: *X*_1_ and *X*_2_, respectively. The differential equation for *S*_ext_ therefore reads as follows:
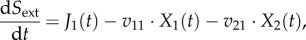
where *v_ij_* is the specific uptake/production rate of compound *j* by species *i*. At steady state, the temporal change of *S*_ext_ is 0 and the organisms' biomass increases exponentially with their respective growth rates
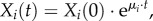
so that one obtains3.1
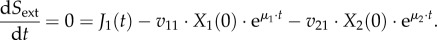
The clearest solution to equation (3.1) can be derived if both organisms grow at the same rate and *J*_1_(*t*) also increases exponentially with that same rate
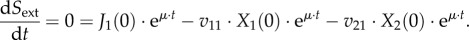
Normalizing this expression with respect to the total biomass in the system given by
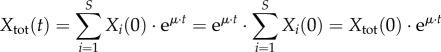
yields
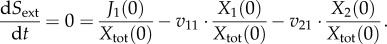


When we introduce
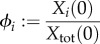
and
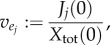
for the relative biomass abundances and the specific environmental exchange fluxes, respectively, we obtain the final expression3.2
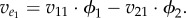
Equation (3.2) shows that biomass abundances have to be taken into account when the balances of external metabolites are calculated. This is a major difference from classical FBA where the relative biomass abundance is always 1 by definition (equation (2.15)). For all internal metabolites biomass abundances do not matter as seen before (equation (2.16)) and cancel out as demonstrated for *M*_11_3.3
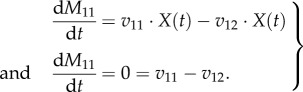


This derivation assumed equal growth rates for both organisms. In many cases, this is the only sensible steady state that can be achieved by communities. For communities in chemostats, equal growth rates are obvious, as the dilution rate sets the steady-state growth rate for each organism independently. In fed-batch growth, however, one might consider organisms with different growth rates, combined with a double-exponential feeding regime to keep the nutrients constant, following equation (3.1). In addition, during batch growth, in analogy with monoculture batch FBA, there may be a window in time where all organisms are in balanced growth, i.e. their internal components are in steady state, even when the external concentrations are not. However, this is provided that there is no cross-feeding present. If the two organisms were to have different growth rates, the one with the highest growth rate would rapidly outgrow the other. If this fast-growing organism is dependent on a factor *D* secreted by the slow organism (as in figure 1), its growth will soon start to become limited by the relatively slow production of *D*, according to the balance3.4
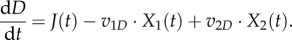
Here, the signs indicate that species 1 consumes *D* produced by species 2, and *J* is the exchange rate of *D*. A small fraction of species 2 will result in slow production of *D*, and, unless *D* is provided from the environment, *D* will decrease until it affects the specific uptake rate of species 1, i.e. *v*_1*D*_. At that point, growth rate will start to decrease for species 1, and the system will settle into a global steady state only when the growth rates are equal. Thus, using steady-state FBA for modelling communities is allowed even if organisms grow at different rates, as long as within that regime the conditions allow constant uptake and production rates. For all practical purposes, that means saturation of the uptake systems. For metabolites that are exchanged such as *D*, saturation is rather unlikely; hence, these models can only represent short snapshots of the system. Either one can resort to dFBA, which glues many such snapshots together (as we discuss later), or one sticks to equal growth rates, as is done in cFBA [39], as discussed in §3.1.

### 3.1. Community flux balance analysis: applications and limitations

cFBA has a number of advantages. First, it provides an unambiguous objective function for all consortium members: the identical growth rate. Moreover, imposing equal growth rates ensures that the relative biomass abundances are constant, and it was shown that these abundances affect the optimal growth rate [39]. Thus, these models predict species abundance ratios, something that can be readily determined experimentally by (metagenomics) sequencing. Steady-state cFBA is, however, mainly applicable to systems in fairly constant environments such as cells grown in the chemostat or cultures used for waste-water treatment.

It is currently unclear whether organisms in a community are actually capable of adjusting their metabolism to operate in an optimal manner: ideally, long-term community chemostats should be studied. In yoghurt fermentations, which are derived from serial transfer of two microorganisms (*Streptococcus thermophilus* and *Lactobacillus bulgaricus*), we do observe biomass abundances close to the predicted optimum (M Hanemaaijer *et al.* 2016, unpublished results). In our view, predictions made by steady-state cFBA should probably be best seen as idealized states that represent potential final states of evolutionary/adaptation processes. Its main applications are the same as for classical FBA in terms of qualitative exploration of medium compositions, metabolic engineering strategies and—most interestingly perhaps—the prediction of essential interactions between species given a certain medium composition. Additionally, cFBA gives an indication of the corresponding optimal relative biomass abundances.

Essential interactions can be identified by optimizing the system's community growth rate and by performing FVA (equation (2.3)) on all the transport reactions of shared metabolites. If the lower and upper values of a flux have the same sign, then it is unidirectional and can therefore be classified as essential. This way, one can easily identify feeding mechanisms and also competition for resources. Please note that in this framework no assumptions are made about the metabolic interactions between species but that these are a direct outcome of the simulations.

If a community of only two species is examined, then the optimal biomass abundances can be obtained by systematically scanning ratios of biomass abundances and calculating the corresponding maximal community growth rate. A plot of the optimal community growth rate versus the biomass ratio then identifies the optimal ratio of biomass abundances [39]. For communities of larger size, a systematic scan is not feasible and therefore optimization methods as gradient descent or evolutionary algorithms can be used with the constraint that the sum of all biomass fractions is 1.

As for classical FBA, steady-state cFBA also comes with certain limitations: absolute metabolite and biomass concentrations cannot be determined but only flux distributions that optimize the community growth rate. It is also important to keep in mind that the results obtained by this method highly depend on the quality of the reconstructed models, and especially their biomass compositions: these define the community growth rate. In addition, the correct identification of possible auxotrophies of individual species during the reconstruction is crucial as these give rise to potential interactions between organisms.

### 3.2. Other approaches to model steady-state behaviour of microbial communities

In most studies that have been published using FBA to study metabolism on a community level, equal growth rates are not assumed. For example, in the first paper on community FBA [36] a syntrophic two-species consortium was examined consisting of *Desulfovibrio vulgaris* and *Methanococcus maripaludis* and an objective function of the type3.5
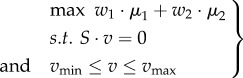
was used for different combinations of weights *w_i_*. Similarly, pairwise interactions of 118 species were investigated and classified as neutral, competitive or cooperative based on a weighted sum of biomass production rates [13]. In [98], growth rates of *Leptospirillum ferriphilum* and *Ferroplasma acidiphilum* are examined in terms of a unidirectional feeding flux from *L. ferriphilum* to *F. acidiphilum* resulting in different specific growth rates. In [99], pairwise interactions of 11 gut bacteria were studied by iteratively fixing the growth rate of one organism and optimizing the other species' growth rate, whereby the resulting Pareto frontiers revealed four different types of interactions. In the OptCom framework, a nested objective function is used whereby a community-level objective is optimized (e.g. maximizing the total biomass of the community) subject to the single-species objectives (e.g. maximizing individual biomass production) [37]. Considering both species-level objective functions and a community-level objective allows cellular and interspecies fluxes to be calculated and trade-offs between species- and community-level objectives to be analysed [37,38,100]. This approach makes no assumption on the relation between individual growth rates that are calculated for each individual species. It is most suitable for well-characterized communities with a defined community objective and, for example, does not include the analysis of communities whose members only compete for resources. However, this approach requires the formulation of a non-convex, bilinear optimization problem that cannot be solved by standard linear programming solvers [37].

Under the constraint that two organisms have to be able to produce biomass at a rate greater than a certain threshold—which allows growth rates to differ—medium compositions that can induce interactions between two species were determined [55]. With this method, known interactions between organisms could be confirmed and new ones predicted, and it allows synthetic communities to be created without modifying the organisms themselves.

These examples show that a lot of valuable information can be gained without assuming equal growth rates, but there are two issues regarding this kind of approach. First, as discussed earlier, these results will describe only a snapshot of the community dynamics, because unequal growth rates will result in rapid changes of shared external metabolites and the biomass abundances of the species. This means that stable relative biomass abundances cannot be inferred when the species' growth rates differ. If this is desired to compare with experimentally determined data, then the equal growth rate constraint needs to be taken into account.

There is a second point to consider when an approach such as equation (3.5) is used, which occurs for communities whose members compete for shared metabolites. If the community species differ in their biomass composition, then a weighted sum of biomass-forming rates will lead to a solution where all available resources are invested in the growth of the organism with the highest biomass yield, i.e. there will be only one biomass formation reaction that carries an actual flux, and which one this is depends on the chosen weights *w_i_*. In other words, all resources will be invested in the least expensive biomass. Technically, this issue can be avoided by assuming equal growth rates [39], by setting lower limits on the growth rates, as in, for example, [55,99], or by coupling the respective transport fluxes to growth rate, as in [101].

One might argue that this problem could be solved by interspecies feeding mechanisms. The reasoning behind this is that then resources cannot only be invested in one organism, but also in the other one because they depend on each other. While this will indeed lead to several metabolically active networks and an exchange of metabolites, still only the biomass-forming reaction of the organism with the highest biomass yield will carry a non-zero flux (if both organisms compete for the same resource and have different biomass yields for this substrate). The network of the other organism will only be used to provide the required metabolite, but resources will not be invested in growth. This is rather artificial as these fluxes require a catalyst—biomass. Coupling of excretion flux to growth rate is one way this problem has been tackled [101].

Besides using explicit constraints on the growth rates, one can also overcome this issue by merging the species' biomass equations into one, so that all the required macromolecules are produced. Instead of maximizing 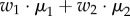 (in the case of a two-species community) one defines according to equation (2.9)3.6
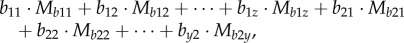
as an objective function where 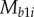 and 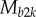 correspond to the biomass components in organisms 1 and 2, respectively. Such a lumped biomass reaction ensures that biomass components of all species in the community are produced. Merging of models into one network, however, should still take the biomass abundances into account for the exchange fluxes; if not it will implicitly assume a species ratio of 1 : 1.

## 4. Dynamic metabolism-based models of microbial communities: dynamic community flux balance analysis

Steady-state cFBA based on balanced growth is well suited to make predictions about communities in fairly constant environments, such as chemostats, regarding their metabolic interactions and optimal relative biomass abundances. However, in batch cultures and many natural communities, organisms are exposed to dynamically changing conditions and can grow sequentially, something this framework cannot accommodate. For such an analysis, dFBA can be used—under simplifying conditions. dFBA was first used to describe the diauxic growth of *Escherichia coli* [85] and has since also been extended to describe community dynamics [14,15,102–107].

dFBA models are similar to FBA models, except that the constraints on the input fluxes are made dependent on extracellular concentrations through kinetic rate expressions. Usually Michaelis–Menten kinetics are used, which are sometimes extended with inhibitory terms [15],4.1
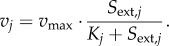


When the *K_j_* parameter is equated to the affinity of the transporter, equation (4.1) describes purely substrate-limited uptake, and thus assumes that the transport step is fully controlling the downstream metabolic pathway. From metabolic control analysis, we know that this is not necessary and in fact not very likely, as control over steady-state flux tends to be distributed [108]. On the other hand, *K_j_* can be interpreted as the Monod constant for growth (which tends to be lower than the *K_M_* of the transporter [109]). In practice, *K_j_* is often fitted to data, and the distinction does not have an effect on the simulations, but it is good to be aware of the underlying assumptions. With the uptake kinetics, a system of differential equations can be defined that describes the dynamics of external metabolite concentrations, the consequent dynamics of uptake rates and, via FBA, biomass formation. Thus, at each time integration step, a linear program computes the growth rate from the uptake kinetics, and both biomass and external metabolites are updated according to4.2
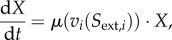
for the biomass, and for the external concentration4.3
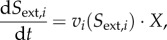
where *v_i_* is the net uptake or production of an external metabolite and the convention in the field is to assign a negative value for uptake fluxes.

**Figure 4.**
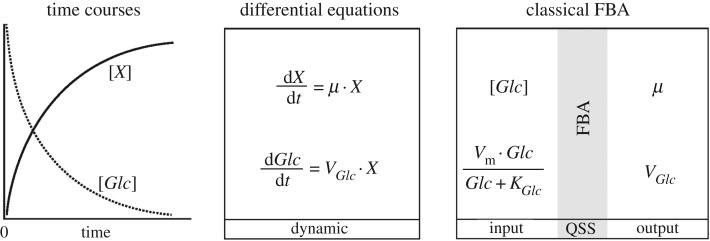
dFBA allows the prediction of time courses for metabolite and biomass concentrations using the quasi-steady-state (QSS) approximation. In this figure, we illustrate the typical results of a dFBA simulation. While a carbon source, *Glc*, is consumed over time, the associated biomass, *X*, increases. The concentration profiles are described by a set of differential equations incorporating the growth rate *μ* and the net uptake rate for *Glc*, *V_Glc_*. These rates are determined using classical FBA whose constraints are dynamically calculated, using Michaelis–Menten kinetics.

One important assumption in dFBA is that the time constants related to intracellular dynamics are much smaller than those that describe changes of external concentrations. This is the basis for the quasi-steady-state (QSS) approximation inherent in the approach. The assumption that metabolism is in QSS during the relatively slow medium transitions, invoked by the cell's own metabolic activities, is very reasonable and generally accepted in the field. However, for adaptations that require gene-regulatory processes that may act at comparable time scales to the environmental changes, this does not apply, and so cells need not to be in balanced growth, in the sense that *all* biomass components, so also proteins, are in steady state. As long as proteome reallocations are not explicitly modelled in genome-scale metabolic models—but they will be in the future—this complication is not an issue. The concept of dFBA is summarized in figure 4.

Extending dFBA to communities is then relatively straightforward: one defines input fluxes for which kinetic expressions are required, and solves the set of differential equations, using FBA for each organism individually. However, here we also face a challenge in the interactions between the species, and different solutions that researchers have suggested.

### 4.1. Dynamic community flux balance analysis: applications and limitations

dFBA has been proven to be a very useful tool to analyse metabolism on a community level [14,15,102–107,110]. It allows communities where species grow in succession to be modelled, which cannot be achieved by steady-state cFBA. Multi-species dFBA was first used to describe the competition between *Rhodoferax ferrireducens* and *Geobacter sulfurreducens* in an anoxic subsurface environment leading to predictions about the conditions under which one can outcompete the other [102]. Later on, this approach was applied to a synthetic community consisting of *E. coli* and *Saccharomyces cerevisiae* that—in this study—exclusively consume xylose and glucose, respectively, to engineer a co-culture system in which both sugars are consumed simultaneously [104]. Chiu *et al.* [111] combined FVA and dFBA to examine 6670 two-species communities consisting of 116 species regarding metabolites that can be produced in a community, but not by a single species growing on the same medium. They showed that emergent biosynthetic capacity occurs in most of the communities, and in two phases: once the two organisms are introduced into the same growth medium and when the medium is nutrient-depleted at the end of a growth phase.

In these studies, the important assumption is made that communities are spatially homogeneous. Often, however, natural occurring communities tend to be structured, which can give rise to interesting dynamics [112–114]. This layer of complexity was addressed in [110], where multi-species community dFBA is coupled with diffusion, allowing the analyses of spatio-temporal effects. This framework was used to predict the species ratio to which a community of two and three members converges, which was found to be in agreement with experimental findings. Similar approaches have also been developed for monocultures [115–117].

As for any application of FBA, the choice of the objective function has certain implications. It has been debated for a long time on which level natural selection occurs: on the species or community level [90–97]. Without specific knowledge about the examined community's objective, it is reasonable to assume that each member of a community attempts to maximize its own growth rate. This is indeed the assumption made in the dynamic multi-species metabolic modelling (DMMM) framework [102], which was the first that allowed dFBA to be performed on a community level.

One very interesting—and challenging—consequence of optimizing organisms individually is that one needs to hardwire the interactions between the organisms, as they cannot be predicted. The reason for this is that at each time instance FBA will predict the currently most optimal flux distribution for growth. It will not predict the suboptimal excretion of a metabolite (e.g. *D*), even though, in the long run, providing *D* to another organism would provide a growth benefit later on. The models cannot predict the optimal strategy over a longer period of time, only at each time instance. If another organism is auxotrophic for this compound *D* and it is not provided in the medium, the growth dynamics of this organism cannot be described without explicitly setting the model up for this purpose. In contrast, in steady-state cFBA, this interaction would show up without explicitly enforcing it.

The d-OptCom framework [107] tries to capture this kind of behaviour by using not only fitness functions of individual species, but also a community-level objective. Using this, the authors claimed to be, indeed, able to describe interactions that could not be modelled using DMMM [107]. Obviously, this approach assumes that a community-level objective function exists, which does not necessarily have to be the case. Moreover, owing to the nonlinear nature of this optimization problem, it is unclear whether this approach will scale—from a computational point of view—when larger communities are examined.

Scaling up to more complex communities is, in fact, a general problem also for dFBA. The extension to larger naturally occurring communities is non-trivial: sequencing and reconstructing metabolic models—including the determination of the respective biomass compositions—for a representative amount of species is, as for all FBA approaches, very time-consuming. It is however important to have good reconstructions as they determine the inference of the interactions between microorganisms. Moreover, as all the interactions ultimately require some kinetic description of the input fluxes, with increasing size of the community, also the number of parameters required to model exchange rates will increase considerably, making it computationally harder to integrate the system and fit the parameters given experimental data. Not surprisingly therefore, so far, dynamic cFBA has mainly been applied to small well-characterized communities whose members have been sequenced (i.e. genome-scale models exist) and where metabolic interactions are known. In these cases, modelling the community with dFBA provides flux distributions within and between species, and can provide insight into the strength of metabolic interactions that cannot be measured directly.

## 5. Summary and outlook

In this paper, we illustrated and discussed how steady-state cFBA can be derived from classical FBA without any additional assumptions. We made a clear distinction between different types of fluxes—specific internal fluxes, exchange fluxes and fluxes towards biomass—as they have different units that are relevant in a community setting. We concluded that stable biomass abundances in communities can only be modelled if (i) the species' relative biomass abundances are explicitly considered and (ii) all organisms grow with the same growth; this ‘community growth rate’ can then serve as the objective function.

cFBA allows optimal biomass abundances and the associated community growth rate at a steady state of balanced growth to be calculated. Furthermore, this approach can be used to infer essential interactions between species in a certain environment without making any assumption about how and whether species interact. However, the cases in which community members all grow at the same rate may be rather limited because that can only be achieved in fairly stable environments. In natural occurring communities, however, dynamically changing environmental conditions are prevalent which cannot be incorporated into cFBA. Here, dcFBA can be used. This approach is, however, limited in its predictive power of unknown interactions and usually requires that the entire system structure is defined beforehand. This is, in particular, true for mutual interactions based on suboptimal growth strategies, i.e. if the excretion of a by-product comes at a growth cost but would benefit an organism in the long run, as it would allow growth of another organism that conversely provides growth-promoting metabolites. While dcFBA is a very powerful tool to describe small synthetic well-characterized communities, its extension to larger communities is not straightforward, owing to the number of parameters required to model transport rate dynamics and the associated numerical instability. One way to keep the number of parameters in a reasonable range is to use explicit exchange kinetics only for metabolites that are essential for the community to grow optimally. This information could be revealed by performing FVA on the solution computed by steady-state cFBA. This combination of cFBA and dcFBA therefore seems promising.

We still face many challenges in modelling metabolism in communities that go significantly beyond insufficient experimental data or reconstructed genomes. We require advances in how to define the objective function of communities, how to deal with dynamics and subsequent (sub)optimal dynamic strategies within the constraint-based modelling format, and how to incorporate the recent advances made in modelling monocultures [82–84]. It seems clear however that better use of metagenomics data towards understanding ecosystem functioning will require models that incorporate such genomics data and ways to make these models useful.
